# Implementing clinical decision support for reducing women Veterans' cardiovascular risk in VA: A mixed-method, longitudinal study of context, adaptation, and uptake

**DOI:** 10.3389/frhs.2022.946802

**Published:** 2022-09-29

**Authors:** Julian Brunner, Melissa M. Farmer, Bevanne Bean-Mayberry, Catherine Chanfreau-Coffinier, Claire T. Than, Alison B. Hamilton, Erin P. Finley

**Affiliations:** ^1^Center for the Study of Healthcare Innovation, Implementation, and Policy, VA Greater Los Angeles Healthcare System, Los Angeles, CA, United States; ^2^Department of Medicine, David Geffen School of Medicine, University of California, Los Angeles, Los Angeles, CA, United States; ^3^VA Informatics and Computing Infrastructure (VINCI), Salt Lake City, UT, United States; ^4^Long School of Medicine, University of Texas Health Science Center San Antonio, San Antonio, TX, United States

**Keywords:** implementation, adaptation, clinical decision support (CDS), women Veterans, cardiovascular risk (CV risk), mixed methods, longitudinal

## Abstract

Evaluations of clinical decision support (CDS) implementation often struggle to measure and explain heterogeneity in uptake over time and across settings, and to account for the impact of context and adaptation on implementation success. In 2017–2020, the EMPOWER QUERI implemented a cardiovascular toolkit using a computerized template aimed at reducing women Veterans' cardiovascular risk across five Veterans Healthcare Administration (VA) sites, using an enhanced Replicating Effective Programs (REP) implementation approach. In this study, we used longitudinal joint displays of qualitative and quantitative findings to explore (1) how contextual factors emerged across sites, (2) how the template and implementation strategies were adapted in response to contextual factors, and (3) how contextual factors and adaptations coincided with template uptake across sites and over time. We identified site structure, staffing changes, relational authority of champions, and external leadership as important contextual factors. These factors gave rise to adaptations such as splitting the template into multiple parts, pairing the template with a computerized reminder, conducting academic detailing, creating cheat sheets, and using small-scale pilot testing. All five sites exhibited variability in utilization over the months of implementation, though later sites exhibited higher template utilization immediately post-launch, possibly reflecting a “preloading” of adaptations from previous sites. These findings underscore the importance of adaptive approaches to implementation, with intentional shifts in intervention and strategy to meet the needs of individual sites, as well as the value of integrating mixed-method data sources in conducting longitudinal evaluation of implementation efforts.

## Introduction

Computerized clinical decision support (CDS) interventions—tools that combine patient information with medical knowledge to guide clinical decisions (1)—have a well-documented track record of shaping practice and patient outcomes (1, 2). Computerized templates, which are a type of CDS, have been deployed to make evidence-based approaches to care more accessible and convenient, for example by facilitating assessment of risk factors for falls (3, 4), or referral to psychotherapy (5). However, the mere availability of a template doesn't ensure that practitioners will use it (6). One of the few studies to report uptake of a computerized template found that it was utilized 5% of the time (7). For templates to be useful, they must be *used*.

Users must be made aware of the template and its value. It must be accessible and convenient to use. It must be tailored to reflect local clinical context, and its use must be supported by the local clinical and organizational culture (8).

Implementation scientists have understood this for years, which is why so much scholarship in implementation science is devoted to (a) adequately capturing *contextual factors* in a given implementation (9, 10), (b) enumerating and evaluating *implementation strategies* to prevent useful innovations from being ignored (11), and (c) characterizing the nature of *adaptations* made to interventions (12, 13).

Although context is diversely defined, it generally refers to social and organizational factors occurring both narrowly within a site and broadly in the site's ecological setting, and is widely recognized for its potential role in impacting intervention effectiveness (9, 14, 15). As Nilsen and Bernhardsson have written, “Accounting for the influence of context is necessary to explain how or why certain implementation outcomes are achieved, and failure to do so may limit the generalizability of study findings to different settings or circumstances” (9). Contextual factors may include the culture, climate, policy, resources, and readiness for implementation of the practice setting and/or external environment (15).

Implementation strategies, or the techniques used to encourage adoption or implementation of a desired intervention, are likewise a critical element of implementation, comprising the “how to” of efforts to achieve practice change (11, 16). Description and evaluation of implementation strategies is one of the core tasks of implementation science, supporting both replication of effective implementation efforts and progress toward a more generalizable science of implementation (16). Meanwhile, adaptations to evidence-based interventions, and to the implementation strategies used in their delivery, are increasingly recognized as occurring frequently (if not inevitably) in scale-up and spread (17–19). Adaptations pose a provocative challenge for diffusion efforts, as they may be associated with improved or reduced intervention effectiveness, and may similarly increase or decrease likelihood of adoption and sustainment; systematic identification and evaluation of adaptations is therefore a critical undertaking (13, 17, 20).

Studies on computerized templates have often acknowledged the importance of each of these aspects of implementation (contextual factors, implementation strategies, and adaptations) (21, 22), but have rarely examined them directly. This omission is often a byproduct of the methods used to evaluate computerized templates. Implementations of templates and other CDS, when evaluated, are most frequently assessed on the basis of quantitative data alone (23, 24). If qualitative data are collected as part of an evaluation, they are typically limited to reports from users of the tool, with the perspectives and insights of *implementers* not systematically documented or reported. Finally, when qualitative data are collected about EHR-based interventions, they are normatively gathered at one or two timepoints (e.g., at baseline and post-implementation), and are therefore insufficient in their ability to capture longitudinal changes in implementation strategies, intervention adaptations, and contextual factors (25).

To address these gaps, we used a convergent mixed-methods design to explore the implementation and uptake of a computerized template for cardiovascular (CV) risk reduction, with the following research questions: (1) what contextual factors emerged in implementation of the CV template across sites?; (2) how were implementation strategies and aspects of the CV template adapted in response to contextual factors?; and (3) how did context factors, use of implementation strategies, and adaptations coincide with differences in template use across sites and across time?

## Materials and methods

### Evidence-based intervention: The CV template

The CV template was developed in response to evidence of provider-level barriers to reducing CV risk (26). These barriers included time constraints, a lack of awareness of current CV disease prevention guidelines, difficulty interpreting guidelines, difficulty accessing relevant patient data at point of care, and low self-efficacy to counsel patients in behavioral change (26–30). The computerized template was intended to aggregate data relevant to CV risk reduction from multiple places in the EHR, and add patient-reported information collected before the visit to enable more comprehensive screening and facilitate provider-patient discussion about each patient's CV risks and possible action steps. The template was made available for use by any provider at a participating site, and all providers were introduced to the template during a local team meeting.

This work was conducted as part of a multi-component trial in Department of Veterans' Affairs (VA) health care facilities funded by VA's Quality Enhancement Research Initiative (QUERI). The trial, called Enhancing Mental and Physical Health of Women through Engagement and Retention (EMPOWER) QUERI, focused on expanding access to important health services for women Veterans (31).

Our EMPOWER QUERI team implemented the CV template as part of a larger “CV toolkit” to identify and document cardiovascular risk screening across women Veterans and engage women in health behavior change. In addition to the CV template described above, which is the focus of this analysis, the toolkit involved two other components, each of which are described at greater length elsewhere (26): (1) a single-page paper-based *self-screener* completed by patients while waiting for a primary care or women's health visit; and (2) a *facilitated group* for CV goal-setting adapted and gender-tailored from a program (“Gateway to Healthy Living”) developed by the VA's national Center for Health Promotion and Disease Prevention. The template and other components of the toolkit were implemented in the context of a non-randomized stepped-wedge trial aimed at engaging and retaining women Veterans in evidence-based care (31). To maximize the applicability of findings across settings, the trial (EMPOWER QUERI) purposively recruited sites with heterogeneous size and structure, particularly with different models for delivering women's health care (31).

### Baseline implementation approach: Replicating effective programs

Replicating Effective Programs is an implementation framework aimed at tailoring evidence-based interventions for delivery in novel settings and/or to novel populations (32, 33). REP was selected for this project because of its well-established evidence base and its track record of constructive application in VA implementation studies (31, 34). REP follows a phased process in which the existing intervention is packaged for delivery in a new setting (pre-conditions phase), tailored in response to feedback from multi-level stakeholders (pre-implementation phase), implemented using a combination of training, engaging champions, and technical assistance (implementation phase), then further customized and examined for sustainability and potential spread (maintenance and evolution phase) (31, 35). In this study we drew upon REP several times in sequence, with all but the initial “pre-conditions” phase repeated at each site.

### Data collection

Our convergent mixed-method implementation evaluation included two longitudinal data sources, periodic reflections (qualitative) and assessment of template uptake using VA administrative data (quantitative). Qualitative and quantitative data were collected in parallel over the course of the study, then analyzed and integrated as described below.

### Periodic reflections

Periodic reflections are a form of guided discussion with implementation stakeholders frequently used to document the dynamic conditions of implementation, including team activities, interactions with site and other partners, key challenges and events, and adaptations to the intervention and/or implementation strategies (25). We conducted 39 reflections as telephone discussions with the CV template implementation team (the single, central team that initiated the overall project, including the co-PIs and project director). Reflections were conducted approximately monthly over the period before, during, and after implementation of a computerized template for cardiovascular risk at five VA facilities (Oct 2016–May 2020). Because each reflection focused on developments since the prior reflection, with alternating periods of activity and inactivity, duration of the discussions varied with the pace of the project developments (20–60 min). Reflections were facilitated by a PhD-level anthropologist, who documented discussion content in detailed, near-verbatim notes. We linked qualitative analyses with descriptive data on template use across the implementation period at all five facilities.

### Template uptake

We measured template uptake at each site by extracting data from the VA's electronic health record. Template uptake was defined as a percentage: the number of patients for whom a template was initiated by participating providers at each site, divided by the number of patients who were eligible to receive a template in that month (i.e., women Veterans who were seen and who had not had a template previously completed).

### Analysis

**Figure 1:**
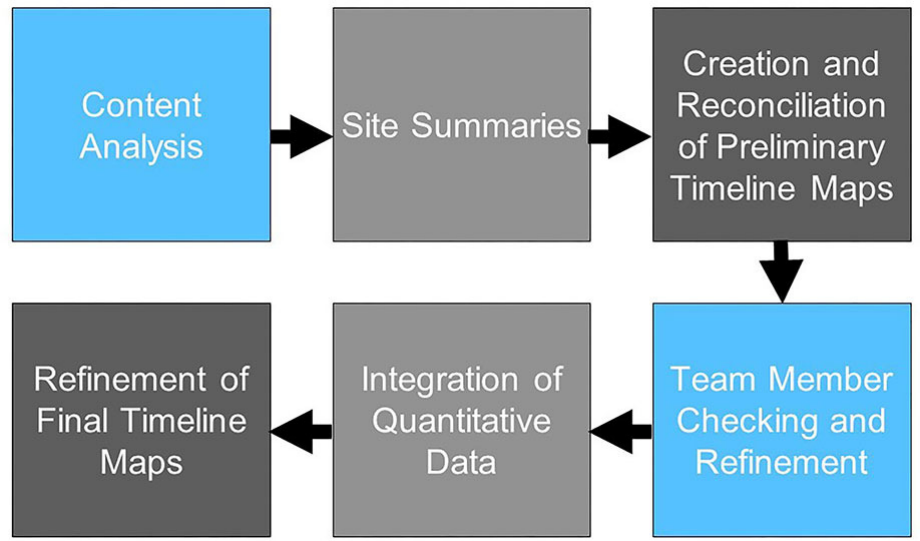
Summary of Data Analysis and Integration.

Our analytic process is summarized in Figure 1. As formal implementation efforts were ending, one investigator (JB) conducted initial review of reflections data to categorize text relevant to identified research questions (e.g., contextual factors, adaptations to intervention, adaptations to implementation strategies); two coders (JB, EF) then reviewed categorized text using a hybrid inductive-deductive content analysis approach. Given the relative dearth of literature identifying high-priority contextual factors in implementation of CDS, we took an inductive approach to contextual factors, independently identifying key themes emerging in the relevant data, then meeting to discuss potential themes and illustrative examples until we achieved consensus for each section of coded text. All text relevant to use of implementation strategies was first coded deductively in accordance with the Expert Recommendations for Implementation Change (ERIC) taxonomy of implementation strategies (11); subsequently, all text descriptive of adaptations to the CV template intervention or implementation plan was coded in accordance with the Framework for Reporting Adaptations and Modifications—Expanded Version (FRAME) (20) or Framework for Reporting Adaptations and Modifications to Evidence-based Implementation Strategies (FRAME-IS) (13), respectively. Following coding, data were reviewed again to create written site summaries identifying: (i) contextual factors, (ii) adaptations to the CV template; and (iii) adaptations to implementation strategies, with approximate dates identified for discrete events. Using these site summaries, two investigators (JB, EF) independently created longitudinal displays of the factors and events most relevant to adoption of the template, i.e., “timeline maps.” The format of these maps, which include a chronological depiction of events and factors grouped into “swim lanes,” builds upon previous applications of systems thinking to program implementation (36). The investigators then met to discuss and reconcile their timeline maps (“initial reconciliation”). The timeline maps were then reviewed by our interdisciplinary team (“member checking”) to verify the accuracy of the maps and identify additional factors viewed as salient by implementation team members, including those who participated in periodic reflections. Once initial reconciliation and member checking were complete and the team reached consensus on the timeline maps for each site, quantitative data on template uptake by month were added to each map.

## Results

The CV template was implemented in three waves across five sites during the period June 2017–March 2020. In sections below, we: (1) describe *contextual factors* emerging across sites during pre-implementation and implementation phases at each site; (2) identify *adaptations* to the CV template and implementation strategies, and; (3) examine *template uptake* and its convergence with contextual factors, use of implementation strategies, and adaptations at each clinic over time.

### Contextual factors

Four key types of contextual factors emerged inductively from our analyses: (1) the pre-existing structure of each site including the model of women's health (WH) care delivery; (2) staffing changes the occurred during implementation; (3) the relative authority of local champions; and (4) leadership external to the clinic.

#### Site structure

Because the intervention was targeted at women Veterans, each site's model for delivering women's health care was a meaningful factor. Three of the five sites were stand-alone comprehensive women's health (WH) clinics, and the other two were general primary care clinics with designated women's health providers (Table 1). Within the three stand-alone women's health clinics, the implementation team aimed to engage the entire clinical staff. At the general primary care clinics, only a designated WH provider and their team nurses and medical/clerical support staff were involved with template use.

**Table 1:** Site profiles.

	**Model of women's health care**	**Local project champion(s)**	**Template users**	**Relationships among sites**
Site A	Stand-alone women's health clinic	Women's health clinic medical director; women's health site clinical lead	All PC teams in the women's health clinic	Shared VA health care system with Site B
Site B	Stand-alone women's health clinic	Women's health clinic medical director; PC team RN	All PC teams in the women's health clinic	Shared VA health care system with Site A
Site C	Stand-alone women's health clinic	Women's health clinic medical director; women's health program manager	All PC teams in the women's health clinic	Sole participating site within their VA health care system
Site D	Women's health embedded in primary care	PC deputy director; designated women's health provider; PC team RN; PC team clerk	One designated women's health PC team	Shared VA health care system with Site E
Site E	Women's health embedded in primary care	PC deputy director; designated women's health provider; PC team RN	One designated women's health PC team	Shared VA health care system with Site D

PC, primary care; RN, registered nurse; VA, Veterans Affairs. VA health care systems typically include multiple local outpatient clinics and other facilities with shared administration, often organized around a primary medical center.

#### Staffing changes

In several clinics, substantial changes in clinic staffing occurred over the course of implementation. At one site (D), the person who had been designated as the sole nurse who would use the template took a leave of absence shortly after implementation. Later, the sole provider designated to use the template left the facility, and then the clinic was shut down amidst the COVID-19 pandemic. Additionally, at site B, extensive staffing changes occurred shortly before implementation, which was noted as a potential impediment: “(Site B) has had some major turnover. Thinking about adding anything to a primary care list under those conditions is not ideal.”

#### Relative authority of local champions

Consistent with REP, the implementation team sought to engage local champions at each of the five sites, but the organizational position and disposition of the champions differed in important ways. At one site (Site C), the key champion had broad authority over the women's health clinic, practiced in the clinic herself, and was unusually supportive and engaged in the implementation of the template.

As the implementation team noted during reflections, “(the champion is) the women's health medical director who said yes (to implementing the template) a year ago. She said, “you're a gift.” She is the person who designed the women's health clinic, including the flow, and hired around that.” The site champion's strong support was reflected in a positive response from clinic members overall at that site. “The reception was overwhelmingly good. They all came right in—when I say all, it was everybody (in the women's health clinic): the front office, the nurses, the providers, the entire team came in and met with us and watched the slide presentation and talked about it. They gave us changes to the wording on the template. They were very engaged and very excited.”

By contrast, while the other four sites each had supportive champions, none of those champions had the same level of local authority (e.g., direct supervisory relationships) or such close working relationships (e.g., long-term co-location) with the clinic staff for whom the template was intended.

#### External leadership

Leadership external to the clinic itself also played a key role, in some cases facilitating rapid change and in others seeming to slow desired progress. In one site, clinic staff requested that the template be accompanied by a clinical reminder to make the template easier to access and prompt its use, only to face continued opposition from a key facility-level leader who objected to a new clinical reminder that was not for all providers. Over the course of 5 months, the implementation team and local providers together made the case that a reminder would be beneficial, and ultimately persuaded the facility-level leader by arguing that the reminder would support progress on high priority performance measures tracked by the facility. Although ultimately successful, resistance from leadership resulted in significant delay in CV template modifications.

In another site, the involvement of a (high-level facility leader) was instrumental in engaging clinical application coordinators (CACs) to execute technical changes to the template. “(The CACs told us) “we're part of (the leader's) group over here,” … So she has a leadership role there…and in the (research unit)…and the school of medicine because she's a provider. She's—besides being incredibly smart—very powerful there, so we're very lucky that she's backing us. And she's been backing us from the first, 5 years ago, but I didn't understand that support until everybody in the clinic mentioned (her)– there's a power there…that's going to help get things done.”

### Adaptations to intervention and implementation strategies

All sites received the phased REP implementation approach, including the strategies of pre-implementation tailoring of the CV template, identifying and engaging champions, and providing ongoing technical assistance during the implementation phase. Over the course of implementation, adaptations were made to both the CV template and to the use of implementation strategies at sites, including both planned changes and changes that were unplanned but emerged as a result of local events and factors occurring at the sites (“responsive”). Table 2 provides a summary of adaptations and the sites where they occurred.

**Table 2:** Adaptations to the CV template and use of implementation strategies.

**Adaptations**		**When the modification was made**	**Planned vs. Responsive**	**Who determined the modification should be made**	**What is modified**	**Nature of modification**	**Goal of the modification**
Adaptations to CV Template	Tailor to local resources	Site A: Pre-imp Site B: Pre-imp Site C: Pre-imp Site D: Pre-imp Site E: Pre-imp	Planned	Implementation team + users	Content	Tailoring	Improve fit
	Re-customization	Site A: Imp Site B: Imp Site C: Pre-imp Site D: Pre-imp Site E: Pre-imp	Planned	Implementation team + users	Content	Shortening; Reordering; Refining	Improve fit, increase satisfaction
	Split template into two (nurse component + provider component)	Site A: Imp Site B: Imp Site C: Pre-imp Site D: Pre-imp Site E: Pre-imp	Responsive	Site lead	Context	Setting and Personnel	Improve fit
	Clinical Reminder	Site A: Imp Site B: Imp Site C: Pre-imp Site D: Pre-imp Site E: Pre-imp	Responsive	Individual practitioners	Implementation	–	Provide prompt
Adaptations to the REP Implementation Approach	Academic detailing	Site A: Imp Site B: Imp Site C: N/A Site D: N/A Site E: N/A	Responsive	Implementation team	Content	Integration of another strategy	Increase provider motivation/self-efficacy
	Creation of cheat sheets	Site A: Imp Site B: Imp Site C: N/A Site D: N/A Site E: N/A	Responsive	Implementation team	Content	Integration of another strategy	Increase provider self-efficacy
	Small scale pilot testing	Site A: N/A Site B: N/A Site C: N/A Site D: Pre-imp Site E: Pre-imp	Responsive	Site lead	Content	Integration of another strategy	Staged implementation

Adaptations characterized using FRAME (20). We adapted FRAME language slightly in characterizing adaptations as planned vs. responsive (rather than the original “reactive”) in order to better reflect the intentional and engaged nature of adaptations made in dialogue with sites. FRAME also specifies the level of the delivery of the adaptation and whether it is fidelity consistent. All adaptations were at the organizational level, and were fidelity consistent (i.e., core elements of the intervention were preserved). Imp: implementation. Pre-imp: pre-implementation.

Planned adaptations of the CV template began with *tailoring to local resources*. Because each VA facility offers a different array of programs for CV risk management, the template was tailored to accurately reflect those resources, allowing providers to make patient referrals appropriate to the local setting. A second planned adaptation of the template focused on *re-customizing* to meet sites' local workflows. The implementation team solicited input from local champions and other template users about the usability of the template and ways to better match the template to local workflows; resulting changes included a reduction in the number of template fields that were mandatory, consolidation of potentially redundant fields describing patients, and other modifications intended to streamline the template.

Interestingly, two unplanned, responsive adaptations of the template emerged from discussions around tailoring and customization. The first of these adaptations involved *splitting the template* into two separate components. A local program champion, in preparation for implementation, noted that the template could be adapted to better reflect the team-based care delivered at her facility. She suggested that the work of entering information from the written screener into the EHR and answering patient questions about the screener could be done by a nurse before the provider arrived to help patients set goals and make referrals to relevant programs. The template was therefore divided into two components to reflect local workflow patterns: (1) a nurse-facing template that mirrored the patient screener, allowing the nurse to enter patient data and document CV risks; and (2) a provider-focused template that encouraged the provider to communicate with the patient about prioritizing CV risks, identify action steps for reducing risks (e.g., smoking cessation), and offer potential referrals to support health behavior change.

A second responsive adaptation occurred following a request that template completion be facilitated by the prompt of an *electronic clinical reminder*. In pre-trial pilot work to develop the template, clinical stakeholders had specifically noted that they were overburdened by clinical reminders and did not want another added (26). As a result, the implementation team was surprised when front-line clinicians at multiple sites requested that the template be facilitated by an electronic reminder. “I think the biggest surprise was that the nurse who does the front end, the one who does the vitals and everything, she looked up and said, “is there any way you could make this a reminder? Because it's easier on us if you just make it a reminder.”” After the reminder was implemented and positively received at one site, it effectively became a site-level “menu option” for the others, all of whom eventually elected to incorporate the reminder. This was accomplished by working with site-level EHR administrators who were able to target the reminder at the site's designated women's health providers.

Finally, adaptations were also made to the planned use of implementation strategies, particularly in the first two sites, where CV template use was slow to get off the ground after launch. At two of the sites, the implementation team conducted *academic detailing*: attending regular clinical meetings and encouraging the use of the template, soliciting feedback about it, and offering strategies for its use. “(Implementation lead) goes to the monthly meetings, so she did that for (site) last month, really pushing to get the trainees to use the template….”

At the same two sites, the implementation lead also worked with clinical champions to develop brief *cheat sheets*, or written instructions that were affixed to clinic computer monitors, to remind and assist providers and staff in using the template.

Finally, at a later site, the implementation team adopted *small-scale pilot testing* in response to a site's concern about expanding template use across the clinic prior to conducting a small trial first. “Their main concern was for the nurses' time in putting the part 1 screener into the template … We decided at the end of the call that we would only have (a nurse) do the template for (a single provider's) patients, and pilot with them first, and then discuss with the other nurses.”

### Template uptake: Site-level implementation

Descriptions below provide a brief summary of overall site-level template uptake, examining the longitudinal course of contextual factors, implementation strategies, adaptations, and implementation progress over time at each site.

#### Site A

**Figure 2:**
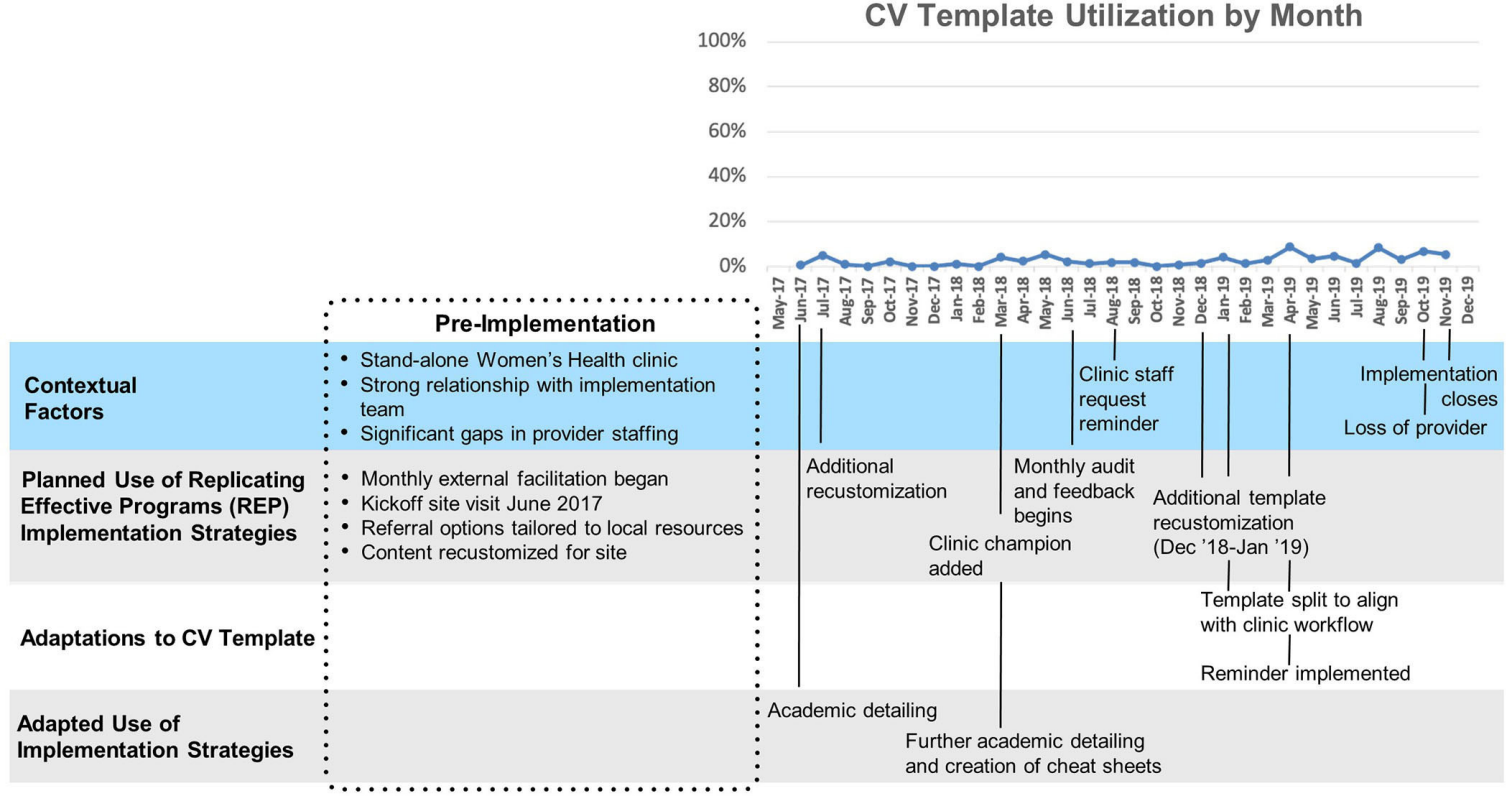
Site A Timeline Map.

Site A (Figure 2) had relatively low overall uptake of the template (Mean 3%, SD 2%). After a ten-month initial period following template launch where uptake remained close to zero, two changes were made: a clinical reminder was introduced and the template was split into a nurse-facing template focused on assessing CV risk, and a provider-facing template focused on goal-setting and referrals. A modest increase in template use was observed immediately following these changes. This site was the first to implement the template and had the longest cumulative exposure to the template.

#### Site B

**Figure 3:**
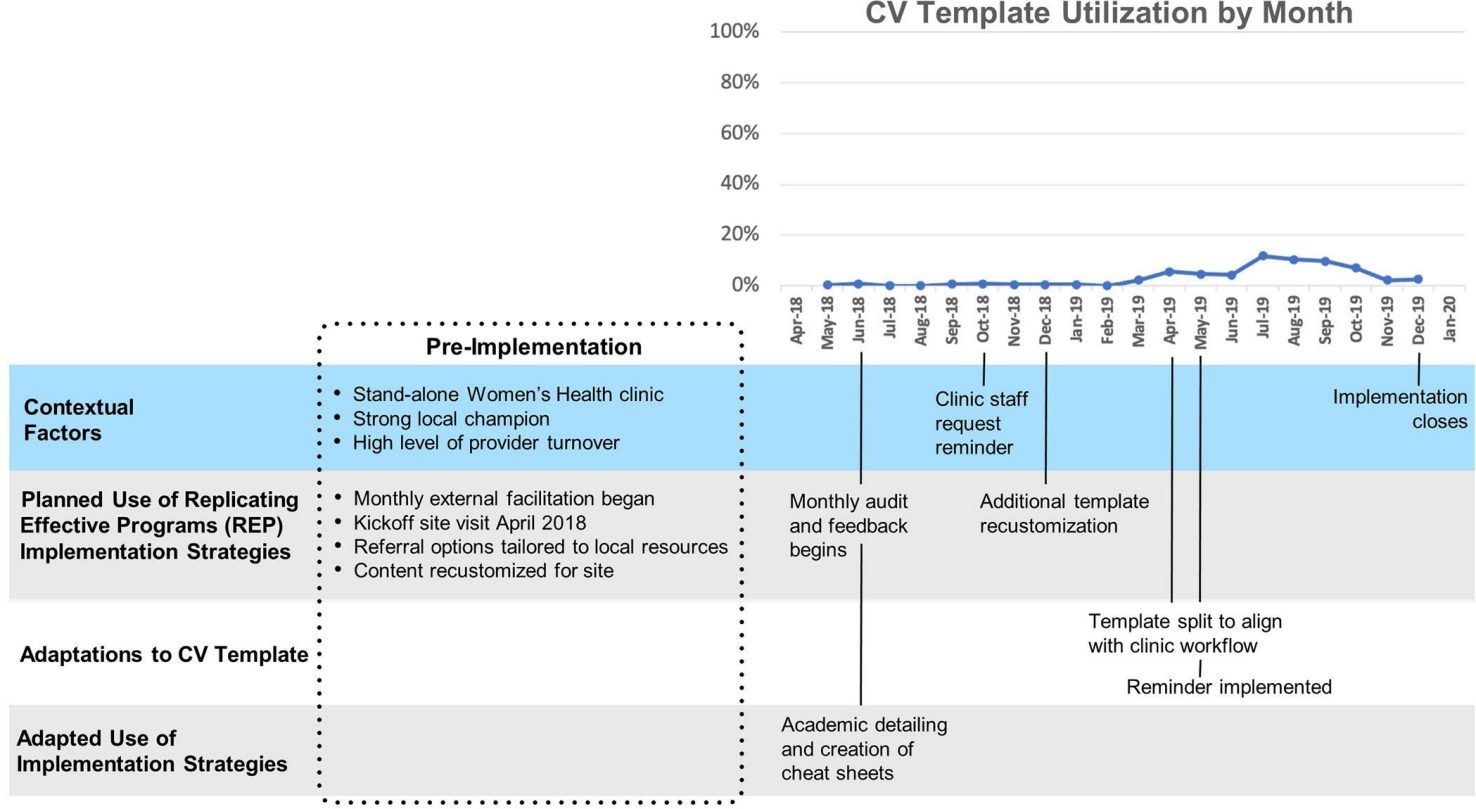
Site B Timeline Map.

Site B (Figure 3) also had low overall template uptake (Mean 3%, SD 4%). Similar to Site A, template uptake at site B was very low until a reminder was introduced and the template was split into two parts, but the modest increase in uptake was temporary. Though staffing in the women's health clinic was relatively stable during the implementation period, substantial turnover had occurred shortly before implementation: “…three providers have changed, three (clerks) have changed, the nurse has changed, a new LVN has changed, two psychiatrists have gone, the others are there but are part-time. (The clinics) have been waylaid by mental health issues from the get-go.” Site B, while geographically distinct from Site A, belongs to the same VA health care system, with shared organizational leadership.

#### Site C

**Figure 4:**
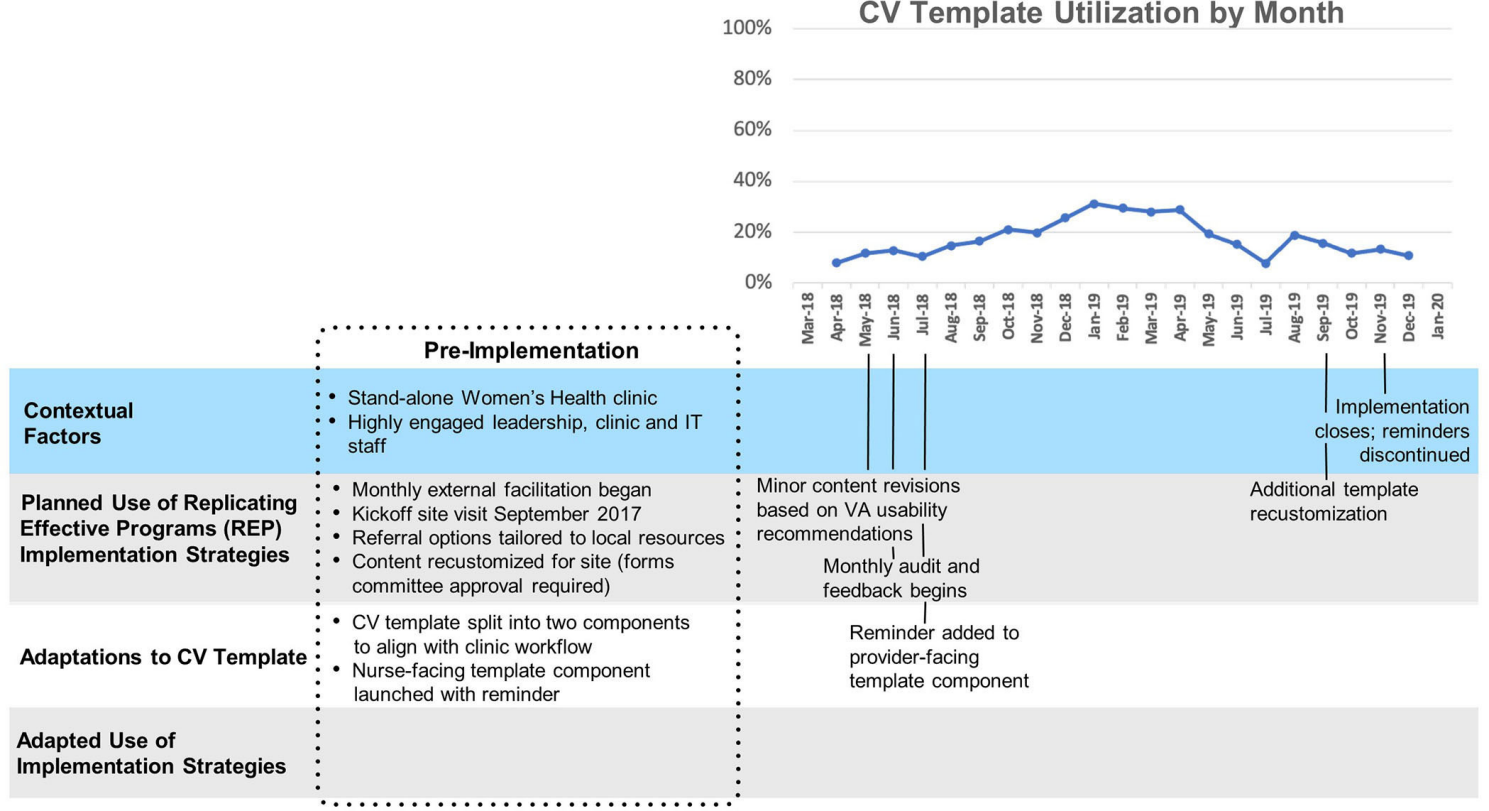
Site C Timeline Map.

Template uptake at Site C (Figure 4) (mean 18%, SD 7%) was consistently higher than at sites A and B, and increased slowly but substantially after technical assistance began and a reminder for the second portion of the template was implemented. “The first screener went on as a clinical reminder immediately, and then this last time they said it would be nice if the provider part came up as a clinical reminder too.” A year after the second reminder was implemented, utilization returned to its pre-reminder level. Of note, splitting the template into two parts and supporting implementation with clinical reminders were innovations/adaptations that emerged first at Site C and later spread to all other sites.

#### Site D

**Figure 5:**
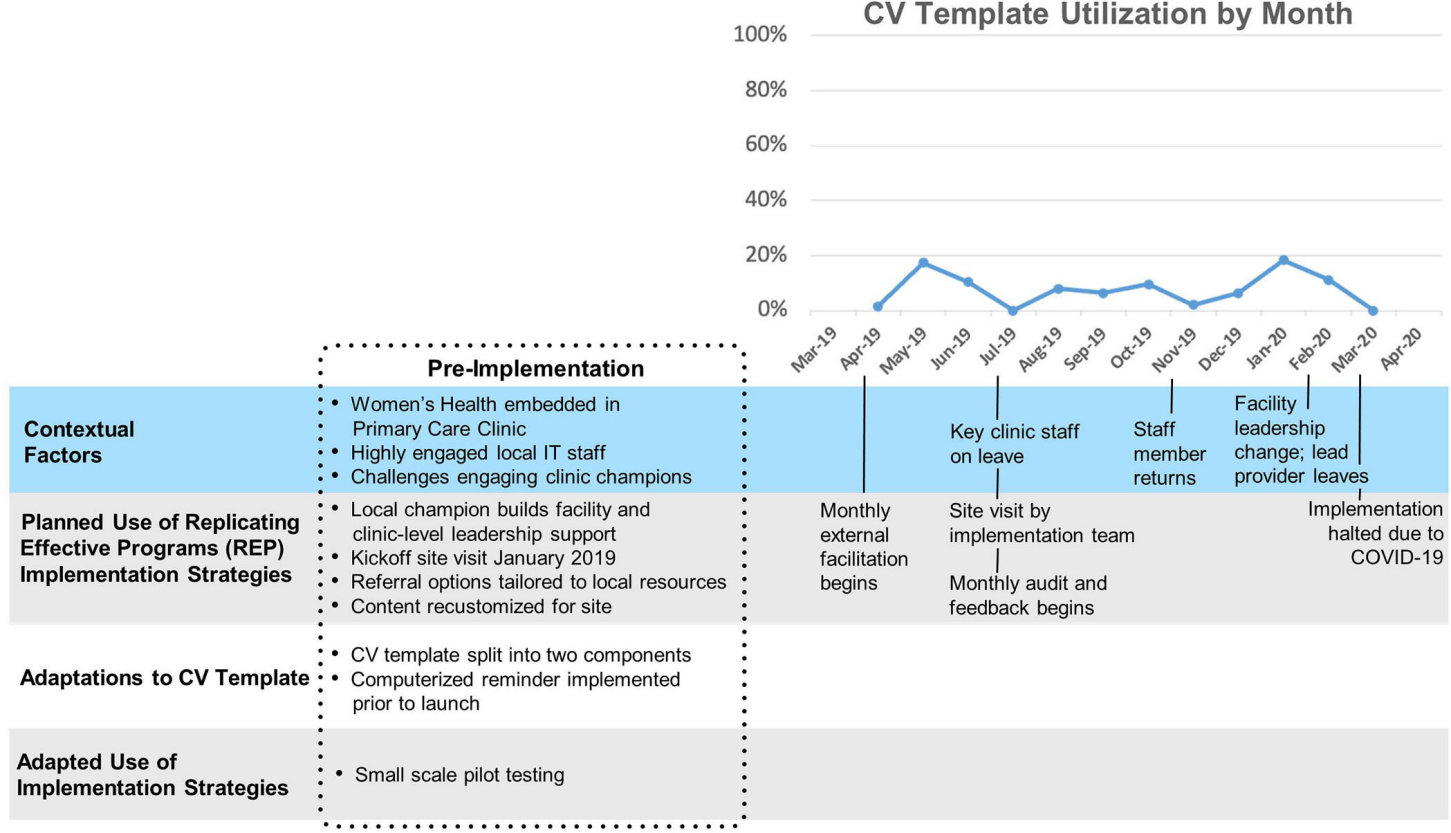
Site D Timeline Map.

Template uptake at Site D (Figure 5) was relatively low (mean 8%, SD 6%). At sites D and E, facility leadership was concerned about the potential time burden that the template would impose and elected to limit the initial implementation of the template to a single primary care team as a small-scale pilot. Template uptake was moderate and highly variable. One of two nurses who had been designated to use the template took a leave of absence shortly after implementation, and her absence was accompanied by a marked decrease in template use. Later, the sole provider designated to use the template left the facility, and the clinic was shut down amidst the COVID-19 pandemic.

#### Site E

**Figure 6:**
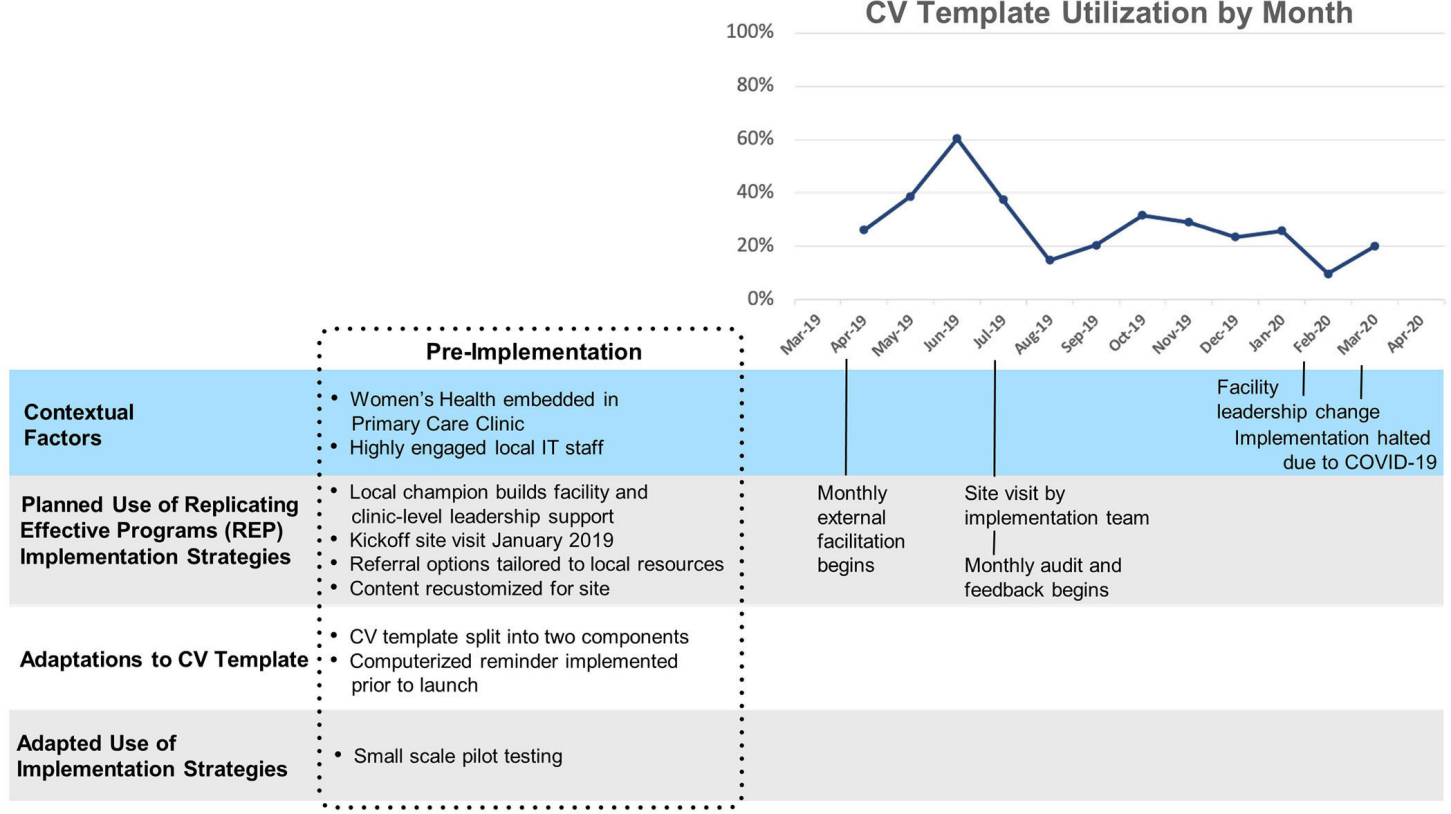
Site E Timeline Map.

The (Figure 6) overall level of template uptake at this site (mean 28%, SD 13%) was substantially higher than at other facilities, and early changes in template use (e.g., a brief spike in uptake above and beyond already-high uptake) did not appear to correspond to events or activities known to the project team. Site E belongs to the same health care system as Site D, and as such shares organizational leadership. Accordingly, the organizational leaders' decision to use small scale pilot testing (with only one care team exposed to the intervention) applied to site E as well as site D. Implementation of the CV template closed ahead of schedule in March 2020 due to COVID-19.

### Template uptake: Cross-site comparison

**Figure 7:**
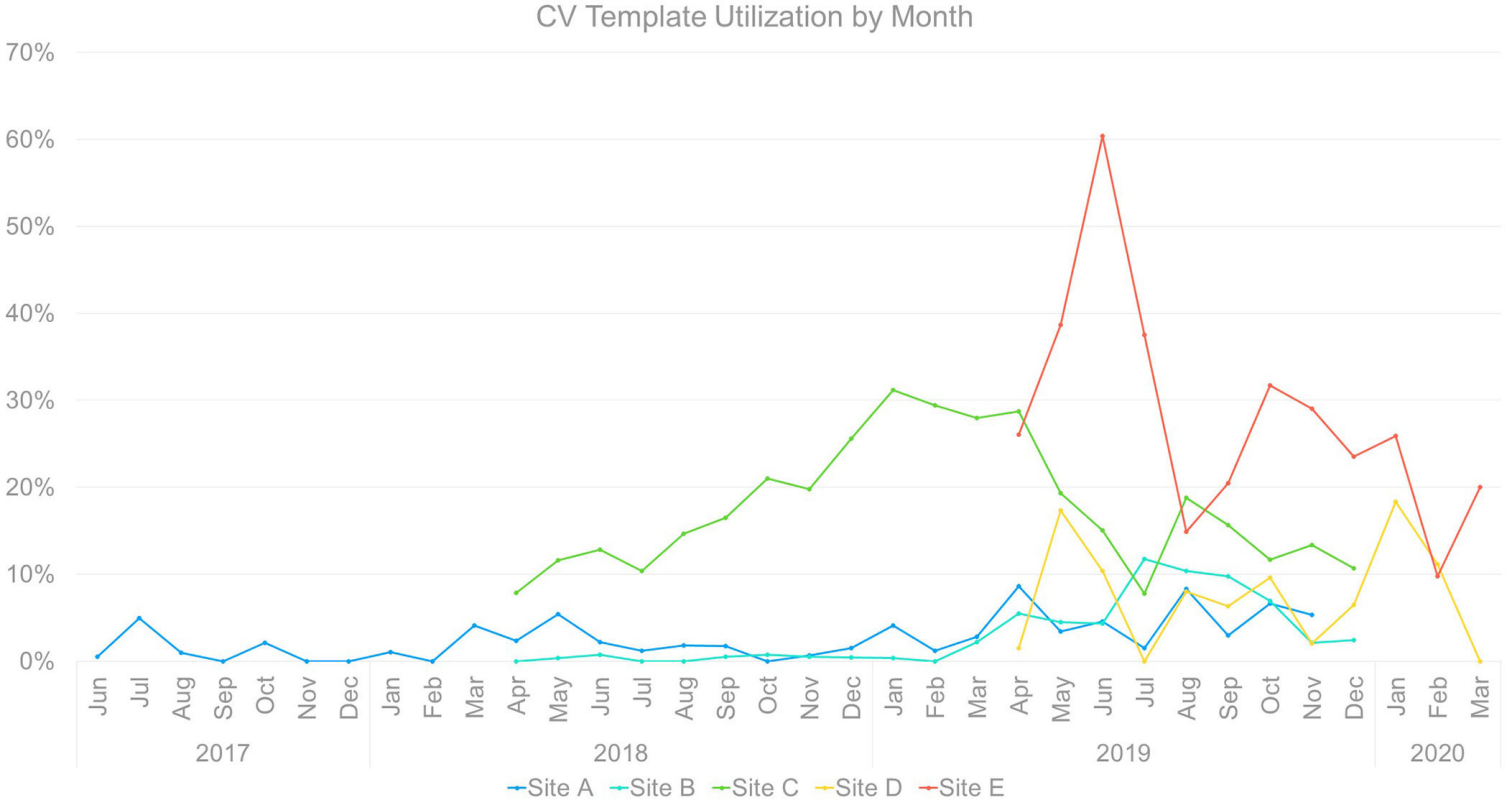
Cross-Site Comparison of Template Utilization by Month.

Notably, there was meaningful heterogeneity of CV template utilization (Figure 7) even among sites within a single organization (VA) and targeting a single population (women Veterans). Heterogeneity occurred across sites in rate of initial uptake, timing and reach of peak uptake, and trajectory of uptake over time.

Implementation across sites occurred in three waves, with one initial site followed by two sites beginning ~10 months later, followed by two additional sites a year later. The timing of waves does not appear to have had significant cross-site effects, as each of the latter waves saw both comparatively high and low performers.

That said, later sites exhibited greater template uptake immediately post-launch, which may reflect incorporation from the beginning of adaptations developed during implementation at earlier sites. All of the sites exhibited variability in utilization over the months of implementation, with apparent convergence between level of utilization and disrupted staffing (reduced template use), overall clinic and leadership buy-in (reduced or enhanced template use), and the onset of COVID-19 and countermeasures (reduced or halted template use).

## Discussion

The current analysis integrated convergent, longitudinal, mixed-method data to examine contextual factors and adaptations associated with implementation of a clinical decision support tool (the CV template) for reducing cardiovascular risk among women Veterans. Our use of timeline maps as site-specific longitudinal qualitative/quantitative displays, along with the use of periodic reflections (25) to capture ongoing insights from implementers, provides a novel approach for assessing implementation of evidence-based interventions and both planned and emergent adaptations. These findings offer a number of insights with implications for design of future CDS implementation and evaluation.

Perhaps unsurprisingly, the contextual factors that emerged as most influential in these findings were related to each site's resources for change (e.g., staffing) and leadership buy-in. Three sites (A, B, D) experienced significant staffing challenges, either immediately prior to implementation launch or during the implementation period, and all saw disappointing template uptake in the months following the shortage. This is consistent with prior studies identifying availability of adequate staff as an important factor shaping capacity for novel change efforts (37–40), particularly given that adoption of new techniques and technologies typically requires additional time and cognitive demand [what Reed et al. (41) refer to as “headroom”] in the period until changes are fully integrated and become routine.

Although champions are widely recognized as a critical component of implementation success (42, 43), these data illustrate the importance of ensuring that site champions have adequate organizational and/or relational authority to support change efforts. The broader importance of leadership buy-in was illustrated in both positive (Site C) and negative (Site A) directions, with leadership support in Site C helping to facilitate adaptation, in the form of implementing clinical reminders to support uptake of the split template, and leadership reluctance in Sites A and B resulting in an extended period of delay before that same adaptation could be put in place. The late-breaking crisis of COVID-19 emerged, too, as an illustration of how acute system shocks can fully derail routine practice, let alone practice change efforts.

These data identified several adaptations to the CV template, taking both planned and responsive forms. Planned adaptations, based in the REP implementation framework, included tailoring and re-customization at each site in dialogue with site-level partners. In exploring the more emergent adaptations we identified, we adapted FRAME language to describe these adaptations as *responsive* (in place of the original FRAME term, “reactive”) to better reflect the intentional and engaged nature of adaptations made in dialogue with sites. These responsive adaptations included both splitting the intervention into two components to allow for a better fit with clinic workflow and integrating computerized reminders to use the template. Both of these adaptations occurred initially in one site but were later offered to and adopted by all four other sites. This provides an excellent example of how adaptations to an evidence-based intervention can be positive and can improve acceptability and feasibility in implementation (17, 18, 44), and may be seen as arguing for the value of formative evaluation in collaboration with implementing sites, particularly during periods of early spread (41, 45, 46). The fact that all sites saw increased use of the CV template following introduction of the clinical reminder underscores the potential value of a “prompt” in achieving consistent behavior change (47–49). The finding that some sites saw a significantly smaller increase than others in template use following introduction of the reminder is consistent with a prior Cochrane review (50), and suggests that even effective implementation strategies and adaptations may be less impactful in settings where context is less supportive of practice change, whether due to inadequate staffing or other challenges (44, 51).

REP as an implementation framework can be viewed as a bundled set of implementation strategies, and in prior work we have noted that REP-specified activities comprise at least 19 distinct implementation strategies (35). Even so, examination of these data allowed for identification of three additional implementation strategies introduced by the implementation team in response to site-level challenges. These included academic detailing and creation of “cheat sheets” for providers in two sites, in an effort to bolster providers' motivation and self-efficacy for utilization of the template, and use of a small-scale piloting approach in another site, where concern was expressed regarding the feasibility of template adoption in a busy clinic. It is worth noting that these strategies emerged in response to local challenges, and were not, in this small sample, typically spread to other sites; moreover, these strategy adaptations were not always successful in achieving a significant increase in template uptake. For both adaptations to the intervention and to implementation strategies, the FRAME and FRAME-IS frameworks provided a thoughtful structure for considering the form and intended function of adaptations, once more demonstrating their analytic utility in implementation evaluation. Use of these frameworks as part of the timeline mapping analysis was particularly valuable in highlighting when adaptations occurred at each site, and whether observable changes in template uptake occurred in subsequent months. Recent contributions to the literature on adaptation in implementation science acknowledge the methodological challenges of assessing adaptations' impact (19, 44, 52), which remain a roadblock to more generalizable understanding of adaptation in the context of implementation (18). These findings and the timeline mapping method provide an example of how innovative use of integrative methods can facilitate evaluation of site-level impact of adaptations over the life course of implementation.

Strengths of this analysis include integration of convergent mixed-method data on template uptake with regular, longitudinal reflections by the implementation team on ongoing events, contextual factors, implementation activities, and adaptations occurring at each site. The timeline mapping approach offers a pragmatic method for examining the longitudinal trajectory of implementation at site and cross-site levels, providing a multi-level perspective on what is happening in implementation, and avoiding the weaknesses of implementation evaluations that rely solely on outcomes gathered at isolated moments in time and may inadvertently obscure key events. In doing so, the use of timeline mapping also answers the call to “embrace a richer and more diverse methodological repertoire when researching complex systems,” (53) by directing attention to learning across sites and the interrelationships among contextual factors and adaptations. Limitations of this approach include the reliance on implementation team perspectives, which may overly bias site-level factors rather than individual provider behavior. Future research should examine integration of individual interviews with providers and clinic staff in order to further assess the accuracy of implementation teams' sensemaking around implementation progress, and to consider the relationships between provider and staff perspectives, implementation team perspectives, and the longitudinal course of implementation uptake as demonstrated by quantitative data (54).

## Conclusions

Heterogeneity in uptake of CDS across sites is widespread but poorly understood. Our analysis used longitudinal joint displays of quantitative and qualitative data to identify key contributors to variable uptake across sites and over time, including contextual factors, active adaptation of the CV template and implementation strategies, and activities and events temporally associated with increases or decreases in template utilization at the site level. These findings underscore the importance of adaptive approaches to implementation, allowing for iterative, intentional shifts in intervention and strategy to meet the needs of individual sites, as well as the value of integrating mixed-method data sources in conducting longitudinal evaluation of implementation efforts.

## Data availability statement

The raw data supporting the conclusions of this article will be made available by the authors, without undue reservation.

## Ethics statement

The studies involving human participants were reviewed and approved by VA Central IRB. Written informed consent for participation was not required for this study in accordance with the national legislation and the institutional requirements.

## Author contributions

JB, BB-M, MF, AH, and EF contributed to the conception and design of the study. CC-C, CT, MF, BB-M, and JB performed statistical analyses. JB and EF performed qualitative analyses. JB wrote the first draft of the manuscript. All authors contributed to manuscript revision, read, and approved the submitted version.

## Funding

EMPOWER was funded by the VA Quality Enhancement Research Initiative (QUERI; grant number 15-272). AH is supported by a VA HSR&D Research Career Scientist Award (RCS 21-135).

## Conflict of interest

The authors declare that the research was conducted in the absence of any commercial or financial relationships that could be construed as a potential conflict of interest.

## Publisher's note

All claims expressed in this article are solely those of the authors and do not necessarily represent those of their affiliated organizations, or those of the publisher, the editors and the reviewers. Any product that may be evaluated in this article, or claim that may be made by its manufacturer, is not guaranteed or endorsed by the publisher.
